# The Rediscovery of POSS: A Molecule Rather than a Filler

**DOI:** 10.3390/polym10080904

**Published:** 2018-08-11

**Authors:** Ignazio Blanco

**Affiliations:** Department of Civil Engineering and Architecture, University of Catania and UdR-Catania Consorzio INSTM, Viale Andrea Doria 6, 95125 Catania, Italy; iblanco@unict.it

**Keywords:** polyhedral oligomeric silsesquioxanes, POSS, composites, thermal stability

## Abstract

The use of polyhedral oligomeric silsesquioxanes (POSSs) for making polymer composites has grown exponentially since the last few years of the 20th century. In comparison with the other most commonly used fillers, POSSs possess the advantage of being molecules. Thus, this allows us to combine their nano-sized cage structures, which have dimensions that are similar to those of most polymer segments and produce a particular and exclusive chemical composition. These characteristics linked with their hybrid (inorganic–organic) nature allow researchers to modify POSS according to particular needs or original ideas, before incorporating them into polymers. In this present study, we first start with a brief introduction about the reasons for the rediscovery of these nanoparticles over the last 25 years. Starting from the form of POSS that is most widely used in literature (octaisobutyl POSS), this present study aims to evaluate how the reduction of symmetry through the introduction of organic groups favors their dispersion in polystyrene matrix without compromising their solubility.

## 1. Introduction

Undoubtedly, the materials that have characterized the last century, were the polymers, due to their ability to provide economic and structural benefits. Continuing in this direction and trying to improve mechanical, thermal and durability properties of polymers by adding various reinforcements to them, material experts are defining the composites as reference materials for the twenty-first century. In the design and assembly of a composite we have to fish in the sea of chemistry, that as we know contains organic and inorganic materials, each with different characteristic properties. Hence, the idea in developing hybrid compounds involves taking advantage of the best properties of each component and trying to decrease or eliminate their drawbacks. Based on this original idea, the prediction is that the defining material platform of the twenty-first century could very well be the hybrid, where two or more components are combined in a single material to give new and previously unattainable combinations of useful properties [1]. In this project, it must be taken into account that design of hybrid materials requires the use of building blocks, in particular those included in the nanotechnology field [1], such as dendritic polymers [2,3,4], carbon nanotubes [5,6,7], graphene [8,9,10], fullerene [11,12,13] and Polyhedral oligomeric silsesquioxanes (POSSs) [14,15,16]. The latter building block is the subject of this review.

**Figure 1:**
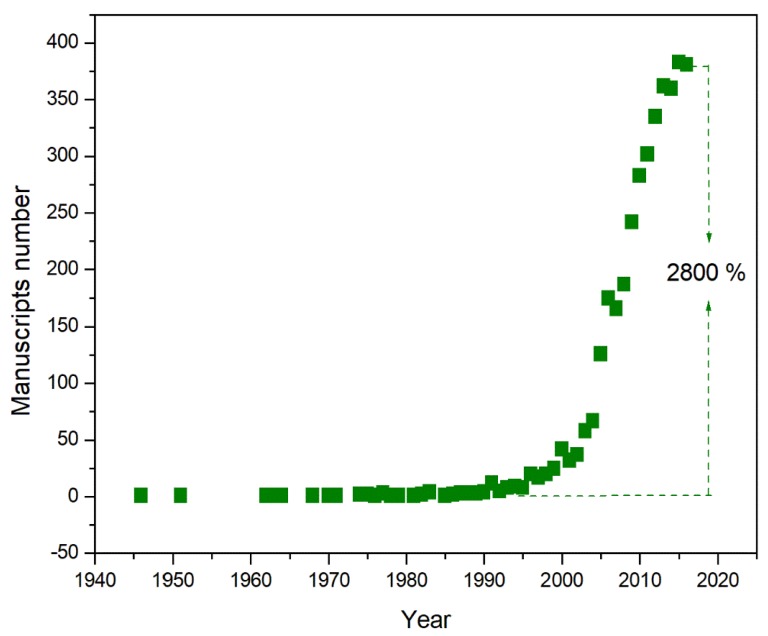
Time evolution of manuscripts with POSS as a topic, which were published on International Journals. Source Scopus.

Unlike the other building blocks, for which few traces are found in the literature before the 1980s, the earliest reports of chemistry relating to silsesquioxanes occurred at the end of the nineteenth century, which were produced by Buff and Wohler [17] and Ladenburg [18]. Just after the end of the second world war, the POSS molecule was first described as we know it today [19]. Considering the small amount of instrumental data available, it is truly surprising how Scott hypothesized not only the oligomer formula but also its structure, surmising that the poor solubility and the tendency to sublimate without fusion were due to its characteristic symmetry. About ten years had to pass before the cube-octameric structure of the molecule was established [20]. In order to appreciate the interest for this topic through the many years, one only has input the keyword “POSS” into the Scopus search engine. After their description as we know today, we only found 85 records in 1946 to 1995, which means less than 2 manuscripts per year in fifty years. Since 1995, we can observe an exponential growth of scientific works concerning POSS (Figure 1).

It increased from an annual average of twenty manuscripts during the five-year period of 1995–2000 to currently reach an annual average of about four hundred manuscripts, with an increase of 2800% starting from 1990 and an increase of 1200% starting from 2000. It is worth noting that in the same period, there has been an overall increase in scientific publications but if we conduct the same operation by entering two other very common search terms, such as Clay or Natural fibers, it can be observed that these terms do not generate a number of manuscripts that is half the number of publications related to the POSS term.

The rediscovery of these materials is certainly due to the work of Feher who set up their synthesis with easily reproducible methodologies [21,22] and Lichtenhan, who understood the infinite potential of POSSs in being able to be mixed with polymers for making hybrid composites [23,24]. Lichtenhan begun to work with POSS at the *Air Force Research Laboratory* at the Edwards Air Force Base (California, USA), where he studied POSS-containing polymers as precursors to hybrid inorganic/organic materials. He subsequently founded the Hybrid Plastics company in 1998, which is actually recognized as the commercial leader for POSSs production. Thus, the most common POSSs are manufactured on a large scale nowadays so that researchers worldwide may purchase these nanomaterials at very competitive prices. Furthermore, the organic nature of the POSSs periphery, which we will analyze in detail later in the text, can be functionalized to generate hundreds of possible compounds. Both purchased and modified POSSs are extremely versatile and may be chemically bonded or physically blended into a companion material, resulting in a hybrid nanomaterial with the combined benefits of POSS and the companion material [1]. This is what we expect of composite technology at its best and it should not be confused with the simple combination of two materials for the sake of it that does not achieve any real design or provide an advantage in terms of material science.

## 2. Experimental

### 2.1. Nomenclature

**Figure 2:**
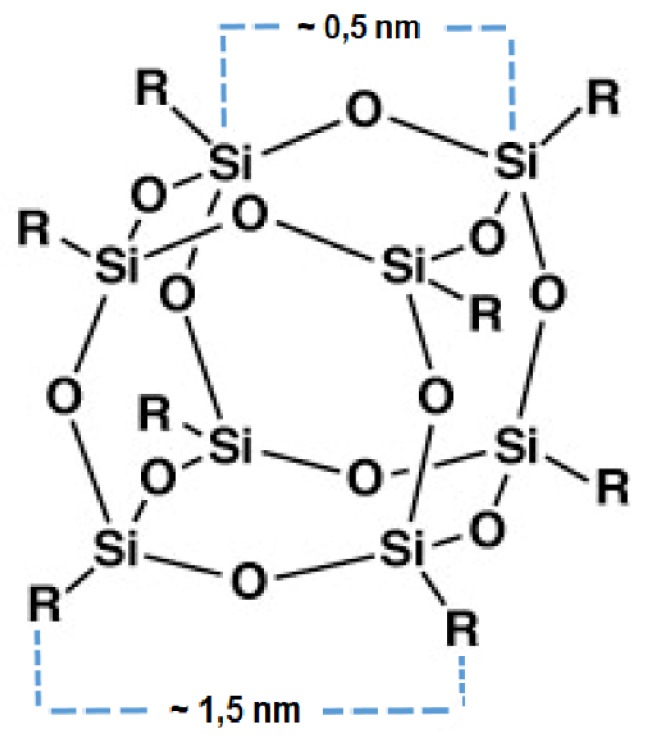
Molecular structure of the classical T8 Polyhedral Oligomeric Silsesquioxanes cage.

The spatial structure of these molecules is not strictly cubic, as first described by Barry et al. [20], because they are actually spherical, hence providing them with the name of Polyhedral. This is because the silicon–oxygen cluster forms a polyhedron not a cube as the silicon–oxygen–silicon and oxygen–silicon–oxygen bonds do not form angles of 90°. The prefix oligo- is for the small number of silsesquioxane units present in the material (sometimes a specific prefix is used to indicate the exact number of repeat units, such as hexa-, octa- and deca, with octahedral silsesquioxane being the most commonly used). Sil- stands for silicon; -sesqui- is added because each Si atom is bound to an average of one and a half oxygens (-ox-); and finally, -ane is used because the Si atom is also bound to one hydrocarbon group. More broadly, silsesquioxanes can be classified into those with un-caged (random, ladder or partially caged) and caged structures [25]. These latter ones with caged structures, which have a general formula of (RSiO_1.5_)_n_, where R is H or an organic group (alkyl, aryl or any of their derivatives), are also known as Polyhedral Oligomeric Silsesquioxanes. These chemicals are composed of a silicon and oxygen cage, which is externally completed by organic groups that are covalently bonded with the silicon atoms. The most common value of n is 8, thus generating a very highly symmetric structure. This is sometimes indicated in the literature with the symbol T_8_, which has a diameter that is usually in the range of 1.5–3 nm (Figure 2).

### 2.2. Materials

The various POSSs were prepared by a corner capping reaction of trisilanol with aryltrimethoxysilane or phenyltrimethoxysilane. Isobutyltrimethoxysilane, cyclopenthyltrimethoxysilane and phenyltrimethoxysilane were purchased from Aldrich Co. (St. Gallen, Switzerland). and used as received. Trimethoxysilane derivatives were prepared from the appropriate Grignard reagent and Si(OCH_3_)_4_ [26,27,28,29]. The different functionalized trisilanol molecules were prepared according to literature methods [30]. The various POSS/PS nanocomposites were obtained by the in situ polymerization of styrene in the presence of different quantities of POSSs. Styrene (Aldrich) was purified by passing it through an inhibitor removal column. We recrystallized 2,2-Azobis(isobutyronitrile) (AIBN) (98% Aldrich) twice from dry ethanol at temperatures lower than 40 °C and not in direct light. Toluene, which was the used solvent, was stirred over calcium hydride for 24 h and distilled in a nitrogen atmosphere.

## 3. Results and Discussion

### 3.1. Incorporation of POSS in Polymer Matrix

POSSs have shown interesting thermal, mechanical, optical and electrical properties [31,32,33,34]. Furthermore, due to their nano-dimensions, they have particularly proven to be the ideal candidates for incorporation into polymer matrices in order to improve their properties. Considering that the size of POSS molecules is usually in the range of 1–3 nm and that a POSS with the very common isobutyl periphery has a diameter of about 1.5 nm [35], these fall under the category of nanocomposites [36,37]. The inorganic silica-based core results in molecule rigidity, thermal stability and resistance to oxidation [38], thus proposing itself as one of the best fillers for polymeric matrices, especially considering its biocompatible nature. Nevertheless, it would be a venial mistake to consider the POSSs as simple fillers. POSS is a molecule rather than a simple filler particle and may be reactive or functionalized to the specific purpose. Thus, the modification of the external organic corona will increase (or decrease) the solubility of POSS in organic solvents [39] and its compatibility with polymers [40,41]. This compatibility and its subsequent capacity to undergo nanometric dispersion is driven by the nature of the organic groups attached to the silicon atoms, which thus strongly affects the properties of obtained nanocomposites [42]. From the available literature, it is also worth noting that when aliphatic groups are attached to the cage POSS, or when long arms are attached to the cage, the solubility in matrix increases, while the presence of short or rigid aromatic groups has the opposite effect [43]. On the other hand, the presence of aliphatic groups in the periphery worsen the thermal properties of the resultant nanocomposite, while the presence of aromatic groups has the opposite effect [44].

### 3.2. The Importance of POSSs Asymmetric Structure in Designing Composites

**Figure 3:**
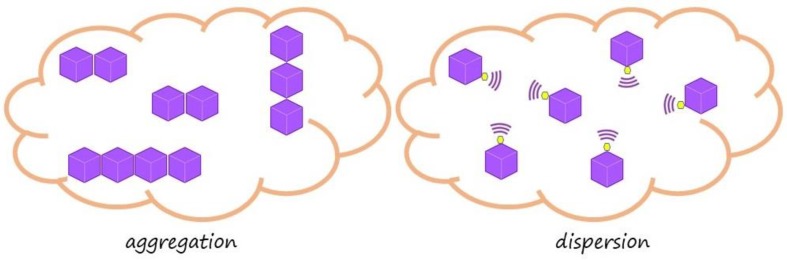
Aggregation vs. dispersion behavior of the different POSS molecules, octaisobutyl-POSS (**left**) and heptaisobutyl-POSSs (**right**), in PS matrix.

Taking advantage of this knowledge, POSSs with seven aliphatic groups and one phenyl group were prepared and used to reinforce Polystyrene (PS), which aimed to obtain nanocomposites with more thermal stability than the matrix. The corner capping reaction of trisilanol with trichlorosilane and/or triethoxysilane [45,46] was carried out to synthesize POSS, while POSS/PS nanocomposites were prepared by the in situ polymerization of styrene with different POSS amounts (3, 5 and 10 wt %) [47]. The PS nanocomposites with heterogeneous POSS showed an extraordinary increase in initial decomposition temperatures (+45 °C about in both oxidative and inert atmosphere) and in degradation activation energy (+40 kJ/mol about) [48,49]. This change was not only in respect to the former polystyrene but overall in respect to the nanocomposite obtained by synthesized styrene with octaisobutyl-POSS (oib-POSS). In particular, among the prepared phenyl heptaisobutyl-POSSs (ph, hib-POSS), the best performance was recorded for the nanocomposites with 5 wt % of POSS. Once the best POSS/PS ratio was established, we needed to explain the considerable increase in thermal stability found for ph, hib-POSS. Homogeneous POSS (with the all the same R groups) can be considered as a symmetric molecule, while heterogeneous POSS (seven same R groups and 1 different) can be considered as an asymmetric molecule. Calorimetric investigation also showed an increase in glass transition temperature (*T*_g_), suggesting a preference in self-regulating phenomena for the symmetric POSS as opposed to the asymmetric ones (Figure 3). A good dispersion is obtained by changing one of the R groups to phenyl, thus allowing the association of the cage with the PS matrix. Such an association does not happen in the aggregation case where all R groups are the same.

Finally, SEM analyses showed the presence of aggregated POSS when oib-POSS is used to reinforce PS, which resulted in a better dispersion of nanoparticles when ph, hib-POSS was used [50,51].

### 3.3. The Influence of POSSs External Groups’ Rigidity on Solubility and Thermal Behaviour

The next step of the research was the synthesis of POSSs with seven phenyl and one alkyl group (ib, hph-POSS) in order to create the relative PS based nanocomposites (ib, hph-POSS/PS). In this case, a thermal investigation was carried out, which showed a dramatic increase in both *T*_5%_ (+116 °C with respect PS and +69 °C with respect to ph, hib-POSS) and *E*_a_ of degradation (+105 kJ/mol with respect to PS and +74 kJ/mol with respect to ph, hib-POSS/PS nanocomposite) values in an oxidative environment. The increasing trend albeit slightly less dramatic was also observed for the degradations performed in an inert atmosphere for both *T*_5%_ (+56 °C with respect to PS and +23 °C with respect to ph, hib-POSS/PS nanocomposites) and *E*_a_ of degradation (+49 kJ/mol with respect to PS). The greater reinforcement, which was due to the replacement of isobutyl with phenyl groups in the POSS corona, was confirmed by the calorimetric experiments carried out to measure the glass transition temperature of the prepared nanocomposites with polystyrene. We observed a *T*_g_ value that was increased by 28 °C compared to that of the oib-POSS/PS nanocomposite and increased by 12 °C compared to that of the ph, hib-POSS/PS nanocomposite. Thus, this supports the hypothesis that the synergistic effect from the introduction of more rigid (in respect to the aliphatic one) phenyl groups with maintenance of the asymmetric structure (by replacing 7/8 organic group at vertices of silicon cage) leads to an increase in the thermal stability of the resultant nanocomposites.

At this stage of the study, the only possibility to further improve the reinforcing effect while still keeping these two points (i.e., the presence of several phenyl groups on the periphery of the POSS molecules and their asymmetric structure) involves obtaining soluble POSSs without a large decrease in the initial decomposition temperature with respect to that of commercial octaphenyl POSS. This can be achieved through the replacement of only one hydrogen atom on one of the eight phenyl groups. Therefore, octaphenyl POSS with small substituents (fluorine and chlorine atoms, methyl and methoxy groups) in the place of one hydrogen atom on one of the eight phenyl groups was synthesized [40] and used to prepare the relative PS nanocomposites. The main concern of using these new slightly modified octaphenyl POSSs focused on their solubility in the PS matrix. These new POSSs were dissolved and the resulting spectroscopic analyses confirmed the structure and properties of the materials so that the subsequent characterization of the chemico-physical properties has been carried out. The *T*_5%_ values of all prepared nanocomposites in both oxidative and inert environments were much higher than those of neat PS and slightly higher than those of ib, hph-POSS/PS nanocomposites (+124–128 °C in air and +69–73 °C in nitrogen with respect to PS; +8–12 °C in air and +13–15 °C in nitrogen with respect to ib, hph-POSS/PS nanocomposites). The *T*_g_ value remained practically the same as that observed for the ib, hph-POSS/PS but this value was higher than that of PS.

### 3.4. The Use of Dumbbell-Shaped POSSs in Reinforcing Polymer Matrix

In recent years, a particular group of POSSs, which are namely “dumbbell-shaped” POSSs formed by an organic group covalently attached to more than one silicon cage, have attracted particular interest. The change in organic bridge leads to a change in geometry, length, rigidity and functionality of obtained POSS, which allows us to subsequently tune its bulk properties [52,53,54]. Polydispersity increased with filler concentration while the d spacing was influenced by phase selectivity and domain–filler compatibility [55].

**Scheme 1:**
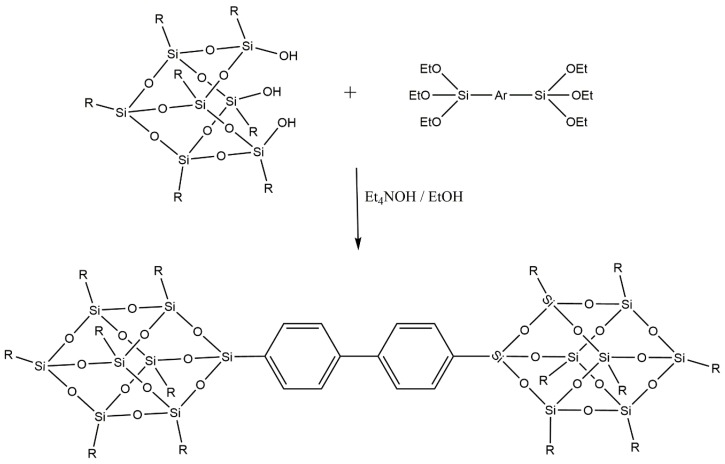
Corner capping reaction of heptaisobutyl-tricycloheptasiloxane trisilanol with triethoxysilyl derivatives for the synthesis of dumbbell-shaped POSSs with an aromatic bridge.

Since we have studied polymer and copolymer systems for sulfonates membranes for several years [56,57], the next step could be the incorporation of POSS into these matrices. Therefore, keeping in mind that the compatibility (i.e., the presence, of groups in the molecular filler that are of the same nature as those present in the polymer chain) between the matrix and filler in these systems is of great importance [58], we do not want to limit the introduction of a simple *T*_8_ cage into PS. For this reason, we synthesized dumbbell-shaped POSSs with a simple and functionalized aromatic bridge using the well consolidated corner capping reaction (Scheme 1) [59].

**Figure 4:**
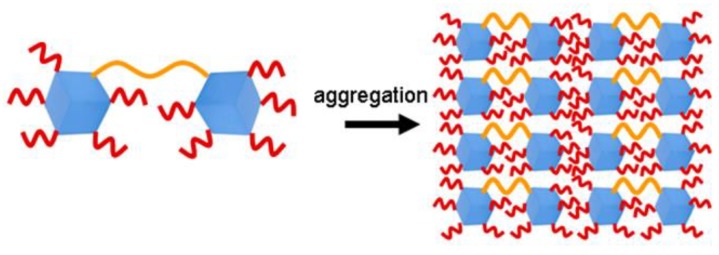
Aggregation phenomena hypothesis for Dumbbell-Shaped POSSs with an aromatic bridge when inserted in PS matrix.

Once synthesized and thermally characterized, a comparison was made with the unbridged systems, dumbbell shaped POSS showed a better thermal stability (*T*_5%_ values ranging from 310 to 410 °C in nitrogen and 290 to 380 °C about in air for the Dumbbell Shaped POSSs vs *T*_5%_ values ranging from 285 to 365 °C in nitrogen and 290 to 380 °C in air for the single cage hepta isobutyl POSS) [46,59]. When inserted in the polystyrene matrix, spectroscopically investigations revealed a poorer dispersion [44] which was attributed to the symmetric structure of the dumbbell shaped molecules that facilitate a POSS aggregation phenomenon (Figure 4). This behaviour led to a maintenance of the thermal stability just seen with the PS nanocomposites reinforced with the single cage hepta isobutyl POSS (*T*_5%_ values of about 290 °C in nitrogen and 260 °C in air) [36].

In order to improve the dispersion of these new very thermally stable nanoreinforcements in the polystyrene matrix, the next design step was to synthesize a series of dumbbell-shaped POSSs with an aliphatic bridge instead of the aromatic one [60]. The result was that the presence in the molecular filler of jointed chains allowed the sufficiently free movement of silicon cages, which increased as a function of the bridge chain length, resulting in a better dispersion of bridged POSSs in the matrix [14].

In terms of the chemico-physical behavior, we found an increase in the resistance to the thermal degradation with respect to the PS reinforced with the POSS bearing an aromatic bridge, which was measured by the initial decomposition temperature and increased as a function of the alkyl bridge length (Table 1).

## 4. Conclusions

Concerning the synthesis of differently characterized POSSs and their incorporation into the polystyrene matrix, it is possible to draw a series of conclusions that can be useful for the designing of nanostructured polymers based on polyhedral oligomeric silsesquioxanes.

By taking for granted that the phenyl groups confer greater thermal stability to the resulting composites, which is supported by the literature [62], this present study confirmed that according to a few studies [63,64], it is preferable to disperse asymmetric POSS molecules in the polymer matrix in order to obtain a nanocomposite with good thermal resistance properties.

Therefore, in the designing of these materials we must ask whether the priority should be the dispersion in the matrix, and then the possibility to opt for the asymmetric un-bridged POSSs, or a higher compatibility with the polymer and then the possibility to opt for the symmetric, but with a bridge that can be functionalized, dumbbell shaped POSSs. An alternative route was proposed, which involved the addition of an aliphatic bridge, which allows the freedom of movement of silicon cages. Due to the spatial blockage, this cannot be achieved with an aromatic bridge.

## Figures and Tables

**Table 1:** Temperatures at 5% mass loss (*T*_5%_) for the various Dumbbell-Shaped POSS/PS nanocomposites with different alkyl bridge length among the POSS cages in static air atmosphere and in flowing nitrogen at 10 °C min^−1^. Values from reference [61].

Nanocomposites	Nitrogen	Air
	*T*_5%_/°C	*T*_5%_/°C
T (CH_2_)_2_ T/PS	284	259
T (CH_2_)_4_T/PS	287	263
T (CH_2_)_6_ T/PS	288	266
T (CH_2_)_8_ T/PS	289	273
T (CH_2_)_10_ T/PS	293	282

## References

[1] Hartmann-Thompson C. (2011). Applications of Polyhedral Oligomeric Silsesquioxanes.

[2] Mezzenga R., Boogh L., Månson J.-A.E. (2001). A review of dendritic hyperbranched polymer as modifiers in epoxy composites. Compos. Sci. Technol..

[3] Díaz I., García B., Alonso B., Casado C.M., Morán M., Losada J., Pérez-Pariente J. (2003). Ferrocenyl dendrimers incorporated into mesoporous silica: New hybrid redox-active materials. Chem. Mater..

[4] Liu H., Guo J., Jin L., Yang W., Wang C. (2008). Fabrication and functionalization of dendritic poly(amidoamine)-immobilized magnetic polymer composite microspheres. J. Phys. Chem. B.

[5] Punetha V.D., Rana S., Yoo H.J., Chaurasia A., McLeskey J.T., Ramasamy M.S., Sahoo N.G., Cho J.W. (2017). Functionalization of carbon nanomaterials for advanced polymer nanocomposites: A comparison study between CNT and graphene. Prog. Polym. Sci..

[6] Vorobyeva E.A., Chechenin N.G., Makarenko I.V., Kepman A.V. (2017). Heat Propagation in Anisotropic Heterogeneous Polymer-CNT Composites. J. Compos. Sci..

[7] Bertolino V., Cavallaro G., Milioto S., Parisi F., Lazzara G. (2018). Thermal Properties of Multilayer Nanocomposites Based on Halloysite Nanotubes and Biopolymers. J. Compos. Sci..

[8] Kuilla T., Bhadra S., Yao D., Kim N.H., Bose S., Lee J.H. (2010). Recent advances in graphene based polymer composites. Progr. Polym. Sci..

[9] Wang H., Hao Q., Yang X., Lu L., Wang X. (2010). A nanostructured graphene/polyaniline hybrid material for supercapacitors. Nanoscale.

[10] Shahil K.M.F., Balandin A.A. (2012). Graphene-multilayer graphene nanocomposites as highly efficient thermal interface materials. Nano Lett..

[11] Wang C., Guo Z.-X., Fu S., Wu W., Zhu D. (2004). Polymers containing fullerene or carbon nanotube structures. Prog. Polym. Sci..

[12] Tenne R., Enyashin A.N. (2014). Inorganic Fullerene-Like Nanoparticles and Inorganic Nanotubes. Inorganics.

[13] Zhao D., Ning J., Wu D., Zuo M. (2016). Enhanced Thermoelectric Performance of Cu2SnSe3-Based Composites Incorporated with Nano-Fullerene. Materials.

[14] Blanco I., Bottino F.A., Cicala G., Latteri A., Recca A. (2013). A kinetic study of the thermal and thermal oxidative degradations of new bridged POSS/PS nanocomposites. Polym. Degrad. Stab..

[15] Carraro M., Gross S. (2014). Hybrid Materials Based on the Embedding of Organically Modified Transition Metal Oxoclusters or Polyoxometalates into Polymers for Functional Applications: A Review. Materials.

[16] Wang K., Pasbakhsh P., De Silva R.T., Goh K.L. (2017). A Comparative Analysis of the Reinforcing Efficiency of Silsesquioxane Nanoparticles versus Apatite Nanoparticles in Chitosan Biocomposite Fibres. J. Compos. Sci..

[17] Buff H., Wohler F. (1857). Ueber neue Verbindungen des Siliciums. Liebigs Ann. Chem..

[18] Ladenburg A. (1873). Ueber aromatische Verbindungen, welche Silicium enthalten. Ber. Dtsch. Chem. Ges..

[19] Scott D.W. (1946). Thermal Rearrangement of Branched-Chain Methylpolysiloxanes. J. Am. Chem. Soc..

[20] Barry A.J., Daudt W.H., Domicone J.J., Gilkey J.W. (1955). Crystalline Organosilsesquioxanes. J. Am. Chem. Soc..

[21] Feher F.J. (1986). Polyhedral Oligometallasilsesquioxanes (POMSS) as Models for Silica-Supported Transiton-Metal Catalysts: Synthesis and Characterization of (C_5_Me_5_)Zr[(Si_7_O_12_)(c-C_6_H_11_)_7_]. J. Am. Chem. Soc..

[22] Feher F.J., Budzichowski T.A. (1989). Syntheses of highly-functionalized polyhedral oligosilsesquioxanes. J. Organomet. Chem..

[23] Lichtenhan J.D. (1995). Polyhedral Oligomeric Silsesquioxanes: Building Blocks for Silsesquioxane-Based Polymers and Hybrid Materials. Comments Inorg. Chem..

[24] Haddad T.S., Lichtenhan J.D. (1996). Hybrid organic-inorganic thermoplastics: Styryl-based polyhedral oligomeric silsesquioxane polymers. Macromolecules.

[25] Blanco I., Abate L., Bottino F.A. (2016). Influence of n-alkyl substituents on the thermal behaviour of Polyhedral Oligomeric Silsesquioxanes (POSSs) with different cage’s periphery. Thermochim. Acta.

[26] Lee J.Y., Fu G.C. (2003). Room-temperature Hiyama cross-couplings of arylsilanes with alkyl bromides and iodides. J. Am. Chem. Soc..

[27] Murata M., Ishikura M., Nagata M., Watanabe S., Masuda Y. (2002). Rhodium (I)-catalyzed silylation of aryl halides with triethoxysilane: Practical synthetic route to aryltriethoxysilanes. Org. Lett..

[28] Weber W.P. (1983). Silicon Reagents for Organic Synthesis.

[29] Manoso A.S., Ahn C., Soheili A., Handy C.J., Correia R., Seganish W.M., Deshong P. (2004). Improved synthesis of aryltrialkoxysilanes via treatment of aryl grignard or lithium reagents with tetraalkyl orthosilicates. J. Org. Chem..

[30] Lichtenhan J.D., Schwab J.J., Reinerth W., Carr M.J., An Y.Z., Feher F.J., Terroba R., Liu Q. (2005). Process for the Formation of Polyhedral Oligomeric Silsesquioxanes. U.S. Patent.

[31] Dou Q., Karim A.A., Loh X.J. (2016). Modification of Thermal and Mechanical Properties of PEG-PPG-PEG Copolymer (F127) with MA-POSS. Polymers.

[32] Mohamed M.G., Jheng Y.-R., Yeh S.-L., Chen T., Kuo S.-W. (2017). Unusual Emission of Polystyrene-Based Alternating Copolymers Incorporating Aminobutyl Maleimide Fluorophore-Containing Polyhedral Oligomeric Silsesquioxane Nanoparticles. Polymers.

[33] Huang C.-W., Jeng S.-C. (2018). Polyhedral Oligomeric Silsesquioxane Films for Liquid Crystal Alignment. Coll. Interfaces.

[34] Li X., Yu B., Zhang D., Lei J., Nan Z. (2017). Cure Behavior and Thermomechanical Properties of Phthalonitrile–Polyhedral Oligomeric Silsesquioxane Copolymers. Polymers.

[35] De Armitt C., Wheeler P. (2008). POSS keeps high temperature plastics flowing. Plast. Addit. Compd..

[36] Blanco I., Bottino F.A., Cicala G., Latteri A., Recca A. (2014). Synthesis and characterization of differently substituted phenyl hepta isobutyl-polyhedral oligomeric silsesquioxane/polystyrene nanocomposites. Polym. Compos..

[37] Kim K., Alam T.M., Lichtenhan J.D., Otaigbe J.U. (2017). Synthesis and characterization of novel phosphate glass matrix nanocomposites containing polyhedral oligomeric silsesquioxane with improved properties. J. Non-Cryst. Solids.

[38] Michałowski S., Hebda E., Pielichowski K. (2017). Thermal stability and flammability of polyurethane foams chemically reinforced with POSS. J. Therm. Anal. Calorim..

[39] Blanco I., Abate L., Bottino F.A. (2017). Mono substituted octaphenyl POSSs: The effects of substituents on thermal properties and solubility. Thermochim. Acta.

[40] Li S., Simon G.P., Matisons J.G. (2010). The effect of incorporation of POSS units on polymer blend compatibility. J. Appl. Polym. Sci..

[41] Blanco I., Bottino F.A. (2015). The influence of the nature of POSSs cage’s periphery on the thermal stability of a series of new bridged POSS/PS nanocomposites. Polym. Degrad. Stab..

[42] Ueda K., Tanaka K., Chujo Y. (2017). Synthesis of POSS Derivatives Having Dual Types of Alkyl Substituents and Their Application as a Molecular Filler for Low-Refractive and Highly Durable Materials. Bull. Chem. Soc. Jpn..

[43] Fina A., Tabuani D., Carniato F., Frache A., Boccaleri E., Camino G. (2006). Polyhedral oligomeric silsesquioxanes (POSS) thermal degradation. Thermochim. Acta.

[44] Blanco I., Abate L., Bottino F.A., Cicala G., Latteri A. (2015). Dumbbell-shaped polyhedral oligomeric silsesquioxanes/polystyrene nanocomposites: The influence of the bridge rigidity on the resistance to thermal degradation. J. Compos. Mater..

[45] Blanco I., Abate L., Bottino F.A., Bottino P., Chiacchio M.A. (2012). Thermal degradation of differently substituted cyclopentyl polyhedral oligomeric silsesquioxane (CP-POSS) nanoparticles. J. Therm. Anal. Calorim..

[46] Blanco I., Abate L., Bottino F.A., Bottino P. (2012). Hepta isobutyl polyhedral oligomeric silsesquioxanes (hib-POSS) A thermal degradation study. J. Therm. Anal. Calorim..

[47] Blanco I., Abate L., Bottino F.A., Bottino P. (2012). Thermal degradation of hepta cyclopentyl, mono phenyl-polyhedral oligomeric silsesquioxane (hcp-POSS)/polystyrene (PS) nanocomposites. Polym. Degrad. Stab..

[48] Blanco I., Abate L., Antonelli M.L., Bottino F.A., Bottino P. (2012). Phenyl hepta cyclopentyl–polyhedral oligomeric silsesquioxane (ph,hcp-POSS)/Polystyrene (PS) nanocomposites: The influence of substituents in the phenyl group on the thermal stability. eXPRESS Polym. Lett..

[49] Blanco I., Bottino F.A. (2013). Thermal Study on Phenyl, Hepta Isobutyl-Polyhedral Oligomeric Silsesquioxane/Polystyrene Nanocomposites. Polym. Compos..

[50] Blanco I., Bottino F.A., Bottino P. (2012). Influence of symmetry/asymmetry of the nanoparticles structure on the thermal stability of polyhedral oligomeric silsesquioxane/polystyrene nanocomposites. Polym. Compos..

[51] Blanco I., Bottino F.A., Cicala G., Latteria A., Recca A. (2016). Synthesis and thermal characterization of mono alkyl hepta phenyl POSS/PS nanocomposites. Polym. Degrad. Stab..

[52] Araki H., Naka K. (2011). Syntheses of Dumbbell-Shaped Trifluoropropyl-Substituted POSS Derivatives Linked by Simple Aliphatic Chains and Their Optical Transparent Thermoplastic Films. Macromolecules.

[53] Araki H., Naka K. (2012). Syntheses and Properties of Star- and Dumbbell-Shaped POSS Derivatives Containing Isobutyl Groups. Polym. J..

[54] Araki H., Naka K. (2012). Syntheses and Properties of Dumbbell-Shaped POSS Derivatives Linked by Luminescent π-Conjugated Units. J. Polym. Sci. Part A Polym. Chem..

[55] Spoljaric S., Genovese A., Shanks R.A. (2012). Novel elastomer-dumbbell functionalized POSS composites: Thermomechanical and Morphological Properties. J. Appl. Polym. Sci..

[56] Abate L., Blanco I., Cicala G., La Spina R., Restuccia C.L. (2006). Thermal and rheological behaviour of some random aromatic polyethersulfone/polyetherethersulfone copolymers. Polym. Degrad. Stab..

[57] Abate L., Blanco I., Cicala G., Recca G., Scamporrino A. (2009). The influence of chain-ends on the thermal and rheological properties of some 40/60 PES/PEES copolymers. Polym. Eng. Sci..

[58] Abate L., Asarisi V., Blanco I., Cicala G., Recca G. (2010). The influence of sulfonation degree on the thermal behaviour of sulfonated poly(arylene ethersulfone)s. Polym. Degrad. Stab..

[59] Blanco I., Abate L., Bottino F.A., Bottino P. (2014). Synthesis, characterization and thermal stability of new dumbbell-shaped isobutyl-substituted POSSs linked by aromatic bridges. J. Therm. Anal. Calorim..

[60] Blanco I., Abate L., Bottino F.A. (2014). Synthesis and thermal properties of new dumbbell-shaped isobutyl-substituted POSSs linked by aliphatic bridges. J. Therm. Anal. Calorim..

[61] Blanco I., Abate L., Bottino F.A., Bottino P. (2014). Thermal behaviour of a series of novel aliphatic bridged polyhedral oligomeric silsesquioxanes (POSSs)/polystyrene (PS) nanocomposites: The influence of the bridge length on the resistance to thermal degradation. Polym. Degrad. Stab..

[62] Tanaka K., Adachi S., Chujo Y. (2009). Structure–property relationship of octa-substituted POSS in thermal and mechanical reinforcements of conventional polymers. J. Polym. Sci. Part A Polym. Chem..

[63] Moore B.M., Ramirez S.M., Yandeka G.R., Haddad T.S., Mabry J.M. (2011). Asymmetric aryl polyhedral oligomeric silsesquioxanes (ArPOSS) with enhanced solubility. J. Organomet. Chem..

[64] Huang M., Yue K., Huang J., Liu C., Zhou Z., Wang J., Wu K., Shan W., Shi A.C., Cheng S.Z.D. (2018). Highly Asymmetric Phase Behaviors of Polyhedral Oligomeric Silsesquioxane-Based Multiheaded Giant Surfactants. ACS Nano.

